# Effect of Drying Methods on Chemical and Sensory Properties of *Cannabis sativa* Leaves

**DOI:** 10.3390/molecules28248089

**Published:** 2023-12-14

**Authors:** Andrzej Kwaśnica, Natalia Pachura, Ángel A. Carbonell-Barrachina, Hanán Issa-Issa, Dorota Szumny, Adam Figiel, Klaudia Masztalerz, Marta Klemens, Antoni Szumny

**Affiliations:** 1 Department of Food Chemistry and Biocatalysis, Wroclaw University of Environmental and Life Sciences, Norwida 25, 50-375 Wrocław, Polandantoni.szumny@upwr.edu.pl (A.S.); 2 Departamento Tecnología Agroalimentaria, Universidad Miguel Hernández, Carretera de Beniel, 03312 Orihuela, Spain; 3 Department of Pharmacology, Wrocław Medical University, ul. Jana Mikulicza-Radeckiego 2, 50-345 Wrocław, Poland; 4 Institute of Agricultural Engineering, Wrocław University of Environmental and Life Sciences, Chełmońskego 37a, 51-630 Wrocław, Poland

**Keywords:** cannabinoids, drying, essential oils, GC-MS, profiling terpenoids, sensory analysis

## Abstract

Hemp is used as a source of fiber, oil and bioactive substances including volatile and cannabinoid-containing substances. This paper presents, for the first time, results on the evaluation of drying methods (convective, vacuum–microwave and combined convective pre-drying and vacuum–microwave finishing drying) of hemp leaves on the qualitative and quantitative changes in secondary metabolites, including essential oils, cannabinoids and sterols. A ranking and descriptive test of hemp leaves was also performed. Drying kinetics was presented using three models, including logarithmic, Midilli and modified Page. The SPME-Arrow technique was used to determine 41 volatile compounds, of which caryophyllene, β-myrcene and α-humulene were dominant in dried and fresh leaves. Regarding the essential oils obtained, 64 were identified, with caryophyllene, humulene epoxide II and limonene being the dominant ones. For preserving the highest amount of oils, the best method was the convective pre-drying followed by vacuum–microwave finishing drying (CD60-VMD) combined method, where the retention of volatile compounds was 36.08%, whereas the CD70 and 240-VMD methods resulted in the highest loss of 83%. The predominant cannabinoids in fresh hemp leaves were CBDA 6.05 and CBD 2.19 mg g^−1^. Drying caused no change in the cannabinoid profile of the plant material. β-Sitosterol, campesterol and lupeol were dominant in the phytosterol and triterpene fractions. No changes in either quality or quantity were observed in any of the variants found.

## 1. Introduction

Hemp (*Cannabis sativa* L.), known as cannabis, is a valued raw material used in the food industry, pharmaceuticals, cosmetics, the paper industry, etc. The most valuable of hemp’s metabolites are cannabinoids, such as cannadbidiol (CBD), tetrahydrocannnabinol (THC), and cannabigerol (CBG) and their acidic forms, essential oils (EOs) and phytosterols [1]. Although the raw material used in the extraction of secondary metabolites is mainly hemp inflorescences, their leaves are an important, albeit underestimated, component of hemp processing. Hemp leaves are widely used industrially as livestock feed, animal bedding textile material, construction and paper components, fuel and biochar substrate [2]. Due to their high mass proportion relative to the inflorescences, despite their lower substance content, they can be a source of valuable phytochemicals such as essential oils or cannabinoids [3]. The extraction of essential oils in the case of raw herbal materials is performed usually from dried material. The effect of the type of drying method of plant material on the profile of secondary metabolites has been proved repeatedly. This is particularly noticeable for essential oils, which, due to their volatility, evaporate very easily during the drying process. Losses of essential oils, depending on the type of plant material, can range from a few to more than 90% of the initial content [4]. Although the most common industrial method of drying herbs is convection drying, it is very rarely optimal in terms of preventing losses of volatile fractions. It should be emphasized here that in the case of industrial drying processes, it is very important to find a compromise between the cost intensity of the process, its efficiency, as well as quality, understood as the content of active compounds and sensory acceptability of the obtained products for consumers. A number of review publications have described how the drying process is a key element of quality assurance. It is a kind of result of two opposing processes, i.e., the loss of volatile components with increasing temperature and the passage of drying time [5,6]. Our experience, as well as that of other groups of researchers, indicates that we cannot predict which of the available drying variants will be the most conservative for the volatile material. Thus, for the volatile compounds present in *Origanum vulgare* [7], the most favorable drying method was vacuum–microwave drying, while for *Thymus vulgaris*, it was a combined 40 °C (pre-drying) and 480 W microwave power [8]. In contrast, for lavender flowers, convection drying at 50 °C had the lowest losses and highest acceptability for the sensory panel [9].

Thus, it seems rational to develop and use a drying technology that will most preserve the volatile compounds present in the plant material [6]. At the same time, the phenomenon of changing the profile of cannabinoids (mainly decarboxylation of acidic forms) during thermal processing and storage of cannabis is described. It has been described that drying of plant material at a relatively low temperature of 50 °C causes a loss of acidic forms of cannabinoids [10,11]. It is known that the therapeutic value of cannabis extracts from the cannabis plant depends not only on the content of various cannabinoids, but also on the ratio of acidic and neutral forms [12]. Hemp leaves are commonly used, also, as an herbal medicinal material or as an ingredient in beverages. They are also applied as a spice or substitute for lettuce for direct consumption. Due to the above, it is important to preserve their desired sensory characteristics during the drying process. Many papers have been published describing the effect of drying on the composition of secondary metabolites of hemp inflorescences [13,14,15].

The presented research shows for the first time what effects various methods of drying Henola hemp leaves, including convection, microwave, and combined, have on the profile of volatile compounds (EOs), phytosterols and cannabinoids. Also, the best drying variant for the sensory characteristics of hemp leaves is pointed out.

## 2. Results and Discussion

### 2.1. Drying Kinetics

**Figure 1:**
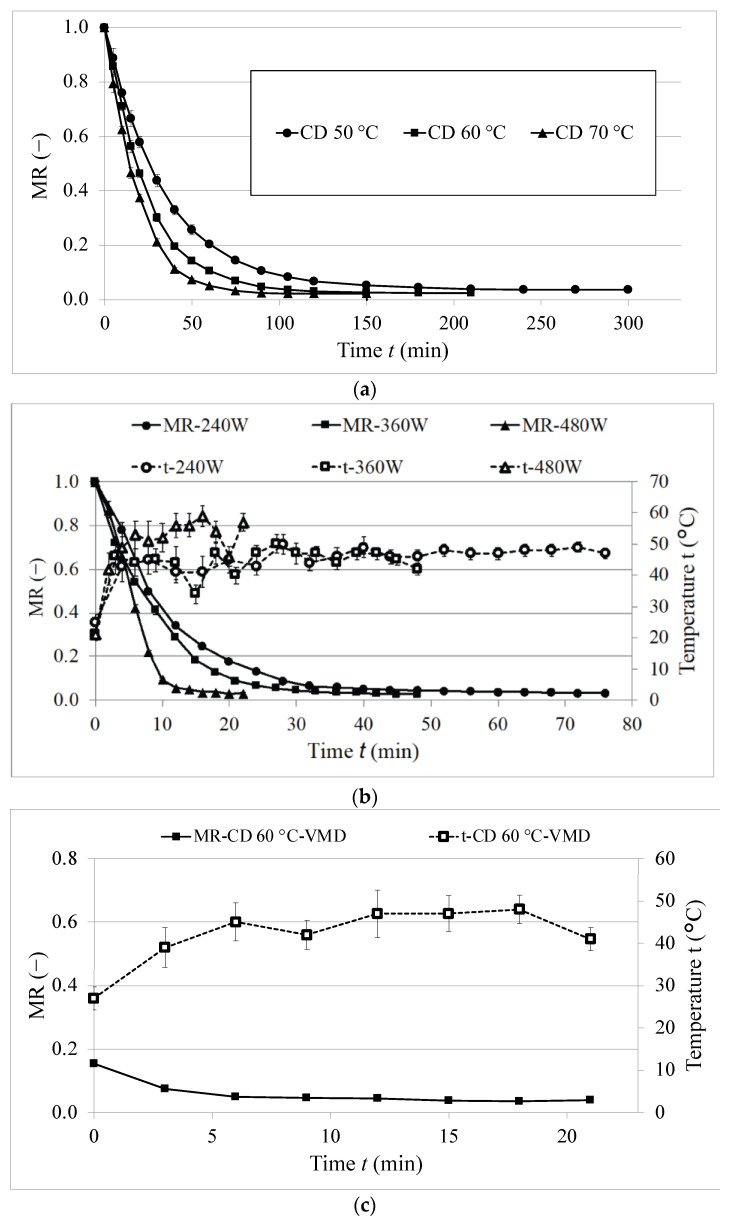
Drying kinetics of the samples treated by (**a**) convective drying (CD), (**b**) vacuum–microwave drying (VMD) and (**c**) combined convective pre-drying and vacuum–microwave finishing drying (CD-VMD).

The drying kinetics of cannabis leaves dried by convective drying (CD) (a), vacuum–microwave drying (VMD) (b) and combined drying (CD-VMD) (c) are shown in Figure 1, while Table 1 presents models used to fit the experimental data.

Three models were used, namely the modified Page, logarithmic and Midilli models. All of them presented a very good fit, determined on the basis of high values of the coefficient of determination (*R*^2^ > 0.9) and low values of RMSE. The modified Page and Midilli models were previously used to describe the drying kinetics of hemp leaves in the study by Chasiotis and Filios [16]. As can be seen in Table 2, VMD480 drying was poorly described by the logarithmic model (*R*^2^ > 0.9527, RMSE = 0.0816), while both modified Page and Midilli models proved to be more suitable to describe more intense drying process, as depicted by the drying kinetics of VMD480. Overall, the Midilli model was selected as the best choice to describe the drying kinetics of hemp leaves.

In all models, the value of parameter *a* was equal to 1, as it shows the starting point for CD and VMD, while in the case of combined drying it was significantly lower, pointing to the beginning of the vacuum–microwave finishing drying. Parameter *k* in the models represented a drying rate. During CD, the *k* parameter was lower than during VMD and increased with an increase in the temperature of the hot air. This is consistent with the studies on the drying of hemp leaves [16]. Higher temperatures during CD accelerated the water evaporation from the surface of the material, leading to a shorter time of drying. An increase in temperature from 50 °C to 70 °C resulted in a significant reduction in drying time, from 300 min for 50 °C to 150 min in the case of 70 °C (Table 3). The VMD method proved to significantly reduce drying time compared to CD despite the power of magnetrons used. Vacuum–microwave drying has been reported for its ability to significantly reduce the drying time compared to other methods such as convective and freeze-drying [17]. This is due to the volumetric heating occurring when the microwaves are applied. As a result, the material is heated from the inside, and an additional pressure gradient between the material and the environment increases the water evaporation, leading to a shorter time for the process. The application of higher power during VMD increases the drying rate, which can be seen as a higher *k* parameter (Table 1). A higher power of magnetrons also increases the temperature of the material, which can reduce the amount of thermolabile compounds in the dried hemp leaves. The obtained results are consistent with the previous studies on hemp flowers [14]. On the other hand, it is worth noting that the highest temperatures of the material were obtained during CD70 (T = 70 °C), and an increase in power during VMD resulted in still lower temperatures of the material. However, the temperatures in the study were measured on the surface of the material, and the actual temperature in the inside might be different. The combination of CD as a pre-treatment with VMD has been reported for its ability to enhance the quality of the products with the following mechanism: pre-drying lowered the unbound moisture of fresh material without any impact on the bioactive compounds of the material, followed by vacuum–microwave drying that brought the moisture content to a certain desired level [18]. In the present study, the application of combined drying reduced the drying time as compared to CD; however, it still resulted in a longer time than when only VMD methods were applied.

According to the decrease in the moisture ratio (Figure 1), it can be observed that only a falling rate period can be distinguished in the convective drying of hemp leaves. This behavior indicates that drying kinetics is controlled by internal liquid diffusion governed by internal heat and mass transfer [19]. This is consistent with previous studies on sage [20] and hemp leaves [16]. On the other hand, during VMD, a constant rate period could be identified, followed by a falling rate period. This can be due to the high initial moisture content in the materials, which led to high microwave absorption resulting in intense water evaporation at the beginning of drying. Then, when the process progressed, the falling rate period could be reported when the drying rate decreased as a result of the dominance of the internal diffusion of bound water, which is consistent with the previous studies [8].

The effects of drying methods and parameters of the process on the color of dried hemp leaves was assessed and is presented in Table 2. Samples treated by CD were significantly brighter (L*) than hemp leaves dried by VMD and CD-VMD techniques. Increases in both temperature during CD as well as the power of magnetrons during VMD led to the increase in the b* parameter, which means a shifting towards yellowness. VMD and CD-VMD had the lowest values for yellowness (b*) compared to the yellowness of the leaves that were treated with the CD treatment. Color degradation commonly occurred during the drying process due to thermal exposure; thus, minimizing thermal exposure during the drying process is expected to lower the color degradation [18]. The same result was observed wherein a higher temperature of CD damaged the color of thyme leaves [21].

### 2.2. Volatile Compounds

#### 2.2.1. HS-SPME-Arrow and Essential Oils Profile of Fresh Hemp Leaves

Volatile organic compounds were analyzed by GC-MS analysis of essential oils and aroma profile analysis using HS-SPME. For the essential oil profile, 66 compounds were determined, 64 of which were identified. On the contrary, 41 compounds were found in the aroma profile, all of which were identified (Table 3). The vast majority of them belong to the group of terpenes and terpenoids.

Table 4 presents the results obtained. For essential oils, the volatile dominating compounds proved to be caryophyllene (33.18% ± 2.76), humulene epoxide II (8.25% ± 0.45), limonene (4.67% ± 0.35), caryophyllene oxide (4.65% ± 0.76), humulene (3.57% ± 0.56), which represented 55% of all EOs. This pattern of terpenes is characteristic of *Cannabis sativa* [22,23]. The aroma profile of the hemp leaves was dominated by (*E*)-β-caryophyllene (30.95% ± 2.35), α-humulene (11.22% ± 0.86), β-myrcene (8.25% ± 1.45), β-selinene (8.95% ± 1.01), limonene (5.47% ± 0.88), α-selinene (7.00% ± 0.89), which together provided 72% of the total fraction of VOCs. A difference in the two profiles was also observed, with β-myrcene, α-humulene, β-selinene and α-selinene only in the aromatic profile (HS-SPME). Such variation in quantitative and qualitative content in the analysis of essential oils and VOCs is a well-known phenomenon. Obtaining essential oil requires the use of boiling water or steam, which completely degrades the glandular trichomes. In contrast, the low process temperature used during the microextraction technique to reach the solid phase causes volatile components that are less shielded by trichomes to volatilize and consequently be absorbed on the CAR/DVB/PDMS fiber.

On the contrary, compounds from the sesquiterpene group and sesquiterpenoids appeared significantly more in EOs, and compounds from the cannabinoid group were also observed: CBD (14.78% ± 1.21), cannabidivarol (0.13% ± 0.03), Δ^8^-THC (0.09% ± 0.02), CBC (0.27% ± 0.06) and Δ^9^-THC (0.14% ± 0.04). Comparing the two profiles, a similar α-pinene content (HS-SPME 2.24% vs. EOs 2.35%) was also noted, as well as a 15-fold higher terpinolene content in the aromatic profile than in the EOs profile and a 10 times higher humulene epoxide II content in the EOs than in HS-SPME. Absent in the volatile fraction analyzed by the SPME technique of cannabinoids, most sesquiterpenoids and the diterpenoid phytol are caused by their very low volatility. Only the work of Woźniczka et al. [24] observed the possibility of determining cannabinoids in biological matrices by SPME methods, but the authors there adsorbed aromatics directly into the liquid matrix.

No work describing the aromatic profile made with HS-SPME for hemp leaves has appeared so far. However, for oils, De Vita et al. [25], in Futura 75 hemp leaves, showed the presence of β-caryophyllene (13.82%), humulene (5.33%), caryophyllene oxide (5.7%) and bisabolol oxide (5.12%) as the main constituents of the essential oil. However, for cannabinoids, CBD was by far the dominant one, with 28.5%, twice that obtained in our study, which may be due to the variety of hemp used. Nagy et al. [26] defined the oil yield in leaves as 0.06%, which is more than 2.5 times lower than the data we obtained (0.16%). Regarding the chemical composition of the essential oil from hemp leaves, caryophyllene was also found to be the main constituent, with a percentage of 28.5%, which is in line with the result obtained by us. However, Nagy et al. [26] in their work obtained a higher humulene content (9.3%) and a lower humulene epoxide II content (1.2%). As far as cannabinoids are concerned, CBD was also the main component, and its variance (11.2%) is analogous to the content in the table below.

**Table 3:** Aroma profile (HS-SPME) and essential oils profile of fresh hemp leaves.

Compound	RI Exp. ^1^	RI Adams ^2^	RI NIST20 ^3^	HS-SPME Content % ^4^	Essential Oil Content % ^4^	Identification ^5^
Hexanal	802	801	800	0.29 ± 0.12	Nd ^6^	MS, RI, AS
3-Hexenal	804	794	810	nd	1.23 ± 0.09	MS, RI, AS
2-Hexenal	851	855	851	1.98 ± 0.27	0.74 ± 0.07	MS, RI, AS
3-Hexen-1-ol	818	859	857	0.81 ± 0.09	nd	MS, RI
*cis*-2-Hexen-1-ol	840	867	868	0.32 ± 0.03	nd	MS, RI
Santolina triene	893	908	908	0.24 ± 0.05	nd	MS, RI
*n*-Heptanal	904	895	901	nd	0.10 ± 0.02	MS, RI, AS
Artemisia triene	924	929	921	nd	0.34 ± 0.10	MS, RI
α-Pinene	939	939	937	2.24 ± 0.21	2.35 ± 0.32	MS, RI, AS
Camphene	944	954	952	0.26 ± 0.07	0.28 ± 0.09	MS, RI, AS
Benzaldehyde	946	960	962	0.34 ± 0.04	0.53 ± 0.12	MS, RI, AS
Sabinene	962	975	974	1.08 ± 0.12	nd	MS, RI
1-Octen-3-ol	969	979	980	0.28 ± 0.07	nd	MS, RI, AS
β-Pinene	977	980	981	nd	0.83 ± 0.15	MS, RI, AS
5-Hepten-2-one, 6-methyl-	978	985	986	0.34 ± 0.04	0.43 ± 0.09	MS, RI, AS
3-Octanone	980	983	985	nd	0.16 ± 0.07	MS, RI, AS
β-Myrcene	982	990	991	8.25 ± 1.45	nd	MS, RI, AS
2-Pentyl-furan	990	991	993	nd	1.09 ± 0.05	MS, RI, AS
δ-2-Carene	1000	1001	1000	nd	Tr ^7^	MS, RI
α-Phellandrene	1004	1002	1005	0.29 ± 0.09	0.30 ± 0.02	MS, RI, AS
*trans,trans*-2,4-Heptadienal	1011	1007	1012	nd	0.34 ± 0.08	MS, RI, AS
3-Carene	1013	1011	1013	0.25 ± 0.05	nd	MS, RI
Limonene	1018	1029	1030	5.47 ± 0.88	4.67 ± 0.35	MS, RI, AS
p-Cymene	1028	1024	1025	nd	0.09 ± 0.03	MS, RI, AS
Eucalyptol	1036	1031	1032	nd	0.11 ± 0.03	MS, RI, AS
β-*cis*-Ocimene	1030	1037	1038	0.87 ± 0.16	nd	MS, RI, AS
β-*trans*-Ocimene	1042	1048	1049	1.57 ± 0.13	nd	MS, RI, AS
Benzeneacetaldehyde	1050	1051	1054	nd	tr	MS, RI, AS
γ-Terpinene	1052	1059	1060	0.35 ± 0.08	nd	MS, RI, AS
*cis*-Sabinene hydrate	1061	1070	1068	0.28 ± 0.05	nd	MS, RI, AS
*trans,trans*-3,5-Octadien-2-one	1075	-	1073	nd	0.25 ± 0.08	MS, RI
Terpinolene	1082	1088	1088	2.28 ± 0.45	0.15 ± 0.05	MS, RI, AS
Linalool	1095	1096	1099	0.30 ± 0.04	nd	MS, RI, AS
3,5-Heptadien-2-one, 6-methyl-	1097	-	1102	nd	0.16 ± 0.04	MS, RI
*n*-Nonanal	1106	1100	1107	nd	0.20 ± 0.09	MS, RI, AS
Fenchol	1107	1116	1113	0.34 ± 0.07	0.16 ± 0.22	MS, RI
*trans*-Pinene hydrate	1117	1122	1121	0.31 ± 0.08	0.25 ± 0.09	MS, RI
p-Mentha-2,8-dien-1-ol	1139	1137	1123	nd	0.53 ± 0.12	MS, RI
Pinocarveol	1143	1139	1139	nd	0.07 ± 0.06	MS, RI, AS
*cis*-Verbenol	1144	1141	1142	nd	0.07 ± 0.03	MS, RI, AS
Ipsdienol	1147	1145	1147	0.22 ± 0.04	nd	MS, RI
Myrcenone	1150	1149	1145	0.50 ± 0.06	nd	MS, RI
*trans*,*cis*-2,6-Nonadienal	1160	1154	1155	0.44 ± 0.04	nd	MS, RI
*trans*-β-Terpineol	1165	1161	1163	0.33 ± 0.08	nd	MS, RI, AS
Borneol	1169	1167	1169	0.29 ± 0.04	nd	MS, RI
1-Nonanol	1180	1173	1169	0.37 ± 0.03	nd	MS, RI
α-Terpineol	1188	1188	1189	nd	0.33 ± 0.09	MS, RI, AS
Hexyl butanoate	1191	1192	1192	0.31 ± 0.10	nd	MS, RI
Estragole	1197	1196	1196	0.96 ± 0.12	nd	MS, RI, AS
Carvone	1249	1243	1242	nd	0.16 ± 0.04	MS, RI, AS
Geranial	1275	1267	1270	nd	0.08 ± 0.03	MS, RI, AS
Carvacrol	1303	1299	1300	nd	0.07 ± 0.02	MS, RI, AS
Guaiacol	1317	1309	1309	nd	0.23 ± 0.09	MS, RI, AS
Cinnamaldehyde	1330	1331	1327	nd	0.08 ± 0.02	MS, RI, AS
Eugenol	1362	1359	1360	nd	0.22 ± 0.09	MS, RI, AS
Ylangene	1380	1375	1372	0.65 ± 0.06	nd	MS, RI
*cis*-Jasmone	1406	1392	1394	nd	0.09 ± 0.02	MS, RI
β-Longipinene	1410	1400	1405	nd	0.61 ± 0.12	MS, RI
Isocaryophyllene	1419	1408	1406	3.86 ± 0.99	nd	MS, RI
(*E*)*-β-*Caryophyllene	1435	1419	1419	30.95 ± 2.35	33.18 ± 2.76	MS, RI, AS
Humulene	1452	1454	1454	nd	3.57 ± 0.56	MS, RI, AS
Aristolene	1455	-	1455	0.89 ± 0.12	nd	MS, RI
9-*epi*-*trans*-Caryophyllene	1465	1466	1464	nd	0.44 ± 0.11	MS, RI
α-Humulene	1470	1454	1454	11.22 ± 0.86	nd	
γ-Selinene	1478	1479	1479	1.25 ± 0.14	nd	MS, RI
*trans*-β-Ionone	1490	1488	1488	nd	0.88 ± 0.21	MS, RI, AS
β-Selinene	1504	1490	1489	8.95 ± 1.01	nd	MS, RI
α-Selinene	1513	1498	1494	7.00 ± 0.89	nd	MS, RI
γ-Cadinene	1515	1513	1503	nd	0.26 ± 0.06	MS, RI
δ-Cadinene	1524	1523	1522	nd	0.44 ± 0.09	MS, RI
Citronellyl butyrate	1530	1529	-	nd	0.11 ± 0.08	MS, RI
*epi*-Longipinanol	1558	1563	1556	nd	0.84 ± 0.15	MS, RI
*trans*-Nerolidol	1568	1563	1566	nd	0.73 ± 0.13	MS, RI, AS
Caryophyllene oxide	1589	1583	1588	nd	4.65 ± 0.76	MS, RI, AS
Hexadecane	1600	1600	1600	2.31 ± 0.32	nd	
Humulene epoxide I	1603	1601	1604	nd	0.75 ± 0.13	MS, RI
Humulane-1,6-dien-3-ol	1608	-	1619	nd	0.12 ± 0.05	MS, RI
Humulene epoxide II	1609	1608	1606	0.73 ± 0.12	8.25 ± 0.45	MS, RI, AS
Javanol isomer II	1623	-	1622	nd	0.30 ± 0.07	MS, RI
Caryophylla-4(12),8(13)-dien-5α-ol	1638	1640	1640	nd	2.02 ± 0.18	MS, RI
14-hydroxy-*cis*-Caryophyllene	1652	1657	1654	nd	2.71 ± 0.15	MS, RI
*Allo*-Himachalol	1663	1661	1662	nd	1.45 ± 0.13	MS, RI
14-hydroxy-9-*epi*-*trans*-Caryophyllene	1677	1669	1676	nd	3.16 ± 0.21	MS, RI
α-Bisabolol	1688	1685	1687	nd	0.17 ± 0.12	MS, RI, AS
Nootkatone	1812	1806	1811	nd	0.99 ± 0.06	MS, RI, AS
Phytone	1848	-	1847	nd	0.98 ± 0.08	MS, RI
*trans*,*trans*-Farnesyl acetone	1922	1913	1921	nd	0.27 ± 0.05	MS, RI
Phytol	2115	2114	2114	nd	2.33 ± 0.17	MS, RI
Unknown 1 ^9^	2225	-	-	nd	0.08 ± 0.04	-
Unknown 2 ^9^	2364	-	-	nd	0.78 ± 0.11	-
Cannabidivarol	2401	-	2406	nd	0.11 ± 0.03	MS, RI
Cannabidiol	2432	-	2430 ^8^	nd	12.73 ± 1.21	MS, RI, AS
Cannabichromene	2438	-	2440 ^8^	nd	0.23 ± 0.06	MS, RI
Δ^9^-THC	2468	-	2465	nd	0.20 ± 0.04	MS, RI

^1^ Experimental retention index calculated by *n*-alkanes; ^2^ literature index according to Adams; ^3^ literature index according to NIST20; ^4^ calculated by peak area normalization according to internal standard; ^5^ identification method: MS—mass spectra (Adams, NIST20), RI—retention index, AS—authentic standard; ^6^ not detected; ^7^ trace < 0.05%; ^8^ according to NAGY [26]. ^9^—MS spectrum available in Supplementary Data S1.

#### 2.2.2. Changes in Aroma Profile during Various Drying Methods

Changes in the content of the major components selected from the HS-SPME profile were significant for each drying method used in the experiment. Although the first three compounds in Table 4 are monoterpenes, the drying methods had markedly different effects on their content. For β-myrcene, a decrease in content was observed with an increase in convection drying temperature, whereas this relationship was not observed for vacuum–microwave drying. For limonene and terpinolene, on the other hand, the situation is the opposite, as an increase in content was observed with increasing vacuum–microwave drying power. For the sesquiterpene group, particularly for caryophyllene, a decrease in content was observed with increasing temperature and power of the drying methods, wherein the combination method resulted in the highest loss of content of this compound. For the other compounds in this group, a decrease in content was observed for all types of drying.

**Table 4:** Changes in aroma profiles of hemp leaves according to various drying methods.

Compound	Fresh	CD50	CD60	CD70	240VMD	360VMD	480 VMD	CD60/VMD
		Content % ^1^						
β-Myrcene	8.25 ^a,2^	11.96 ^b^	3.95 ^c^	1.62 ^f^	1.19 ^g^	1.30 ^h^	2.14 ^e^	2.47 ^d^
Limonene	5.47 ^a^	5.73 ^b^	1.95 ^e^	2.01 ^e^	0.95 ^g^	1.40 ^f^	4.78 ^c^	3.84 ^d^
Terpinolene	2.28 ^a^	1.32 ^c^	6.33 ^d^	3.34 ^b^	6.38 ^d^	9.53 ^e^	12.29 ^g^	10.36 ^f^
Isocaryophyllene	3.86 ^a^	1.95 ^f^	2.32 ^d^	2.09 ^e^	1.13 ^h^	1.64 ^g^	2.59 ^c^	3.08 ^b^
*(E)-β-*Caryophyllene	30.95 ^a^	18.03 ^d^	14.90 ^e^	13.24 ^f^	21.43 ^b^	20.69 ^c^	9.11 ^g^	7.10 ^h^
α-Humulene	11.22 ^a^	5.50 ^d^	5.26 ^e^	5.04 ^f^	7.45 ^b^	7.16 ^c^	4.11 ^g^	3.92 ^h^
β-Selinene	8.95 ^a^	1.21 ^h^	1.89 ^e^	2.05 ^d^	1.78 ^f^	1.50 ^g^	2.35 ^c^	2.84 ^b^
α-Selinene	7.00 ^a^	1.51 ^g^	2.07 ^e^	2.15 ^d^	1.19 ^h^	1.81 ^f^	2.33 ^c^	2.80 ^b^

^1^ Calculated by peak area normalization according to internal standard; ^2^ values followed by the same letters are not statistically different (Tukey’s test, *p* > 0.05).

So far, one publication has been published on the profile of volatile organic compounds in hemp leaves. Rather et al. identified trans-caryophyllene, α-humulene and α-pinene as the main constituents [27]. Referring to the HS-SPME analysis of cannabis flowers by Cicaloni et al. proved that β-caryophyllene, humulene and selina-3,7(11)-diene were found to be the main constituents in the different cannabis varieties [28]. On the other hand, in flowers dried by Oduola et al., the compounds with the highest content turned out to be β-pinene, α-pinene, limonene, β-myrcene and caryophyllene [13].

#### 2.2.3. Changes in Essential Oils Profiles during Various Drying Methods

The effect of the drying methods used on the essential oil content was significant in all variants. These changes, as well as the individual main oil components, are presented in Table 5. The most favorable drying method in terms of oil recovery proved to be the mixed CD60/VMD method, with an oil recovery of 36.08%. The least effective methods were CD70 and VMD240, with an oil recovery of only 16%. It is also worth noting that the oil recovery values change with the drying methods used and their parameters. For microwave drying, an increase in essential oil recovery was observed with increasing process power, and the difference in recovery between the highest and lowest drying power was 12.59%. With regard to the individual components, particularly for limonene, a relationship was observed; with increasing parameters of the drying methods (temperature for CD, and magnetrons for VMD), the content of this monoterpene decreases significantly. This relationship was also observed for caryophyllene, but only during convection drying. In contrast, an increase in content was observed for the other components (humulene, caryophyllene, caryophyllene oxide, humulene epoxide II, Caryophylla-4(12),8(13)-dien-5α-ol, 14-hydroxy-*cis*-Caryophyllene and 14-hydroxy-9-*epi*-*trans*-Caryophyllene). Due to their very low volatility, in most sesquiterpenoid components belonging to the alcohol group, their share increases once the process temperature increases.

There is not a precedent for work in the literature addressing the drying of hemp leaves and analyzing changes in the composition of essential oils. In contrast, these changes are documented for leaves of other plants. For lemon balm leaves, Argyropoulos et al. [29] noted that for β-caryophyllene, with increasing drying temperatures of 30–60 °C the percentage content also increases, while for caryophyllene oxide the highest content was observed at a drying temperature of 60 °C. Szumny et al. [30], investigating the effect of drying on changes in oil content, proved that the content of *trans*-caryophyllene, caryophyllene oxide and β-caryophyllene decreased by half during the drying methods used (CD, CPD-VMFD and VMD). During hop drying, Rybka et al. [31] documented no change during drying at 40 °C and at 50 °C for compounds such as limonene, α-caryophyllene, β-humulene, caryophyllene oxide, humulene epoxide II.

### 2.3. Cannabinoids

Analysis of cannabinoids in hemp leaves revealed the presence of nine compounds (Table 6). The main compound was found to be CBD (2.19 mg g^−1^) and its acid form, CBDA (6.05 mg g^−1^), as well as Δ^9^-THC (0.25 mg g^−1^) and THCA (0.63 mg g^−1^). Other cannabinoids were detected in concentrations below 0.2 mg g^−1^. This profile is in agreement with other authors, including Azad [32] and Li [33].

Our analyses of total cannabinoid content in fresh and dried material proved that the applied variants did not have a statistically significant effect on the quantitative and qualitative profiles. This is an original finding about cannabinoid stability. According to other authors, in general, the storage process leads in effectiveness, with significant degradation of cannabinoids [34].

Other authors, regarding cannabis inflorescences, noted a decrease in acidic forms during the storage of the material. Meija et al. [10] found that after 8 weeks of storage of *C. sativa* herbs in 40 °C THCA, they degraded from 125 to 20 mg g^−1^, whereas CBDA went from 24 to 8 mg g^−1^. At the same time, the amount of THC increased from 60 to 115 mg g^−1^. The concentration of cannabinodiol increased from 9.1 to 18 mg g^−1^ at the same time. Authors found the same values of the sum of acid and neutral forms of cannabinoids. Jaidee et al. [11] have shown that in a temp. of 70 °C, the acidic form degrades to neutral.

### 2.4. Sterols and Triterpenoids

Analysis of the profile of sterols extracted from hemp leaves revealed the presence of four sterols (Table 7), the dominant being β-sitosterol (534.11 µg g^−1^), followed by campesterol (97.90 µg g^−1^), stigmasterol (44.51 µg g^−1^) and isofucosterol (41.38 µg g^−1^). However, for the triterpenoid profile, three compounds were identified, wherein the dominant triterpenoid was lupeol (97.45 µg g^−1^), while β-amyrin and α-amyrin were at similar levels (65.96 and 61.07 µg g^−1^). The overall content of sterols and triterpenoids in hemp leaves was 0.07% and 0.02%, respectively, according to GC-MS analyses.

The results for total sterols and triterpenoids agree with the findings of Jin et al., who in two articles [35,36] showed a similar correlation, as for sterols and terpenoids it was 0.03–0.05%. The main components of the sterol profile were β-sitosterol, campesterol and stigmasterol. On the other hand, in the triterpenoid profile, friedelin appeared to be the main component, followed by epifriedelanol and β-amyrin [35].

Up to now, the content of sterols and triterpenoids in hemp leaves has not been investigated by comparing the effects of different drying methods. In our study, the drying methods used for hemp leaves did not have a significant effect on qualitative and quantitative changes in the profiles of sterol and triterpenoid.

### 2.5. Sensory Analysis

The ranking test showed that samples dried using CD at 50 °C were equivalent to those taken as the control (fresh). Samples were grouped into two clusters: (i) the first one having a higher intensity of the studied key descriptor, including CD at 50 and 70 °C, VMD at 240 and 360 W; (ii) the second one having a lower intensity, including CD at 60 °C, CD 60/VMD, and VMD at 480 W.

The first comment in this section is that the aroma profile of the dried samples was soft, as represented by intensities of the key descriptors (e.g., hemp leaves-ID, citrus, etc.) having maximum values below three units. Significant effects of the drying treatment on only four attributes were found; these included fresh vegetables, hemp leaves-ID, citrus and balsamic notes.

**Figure 2:**
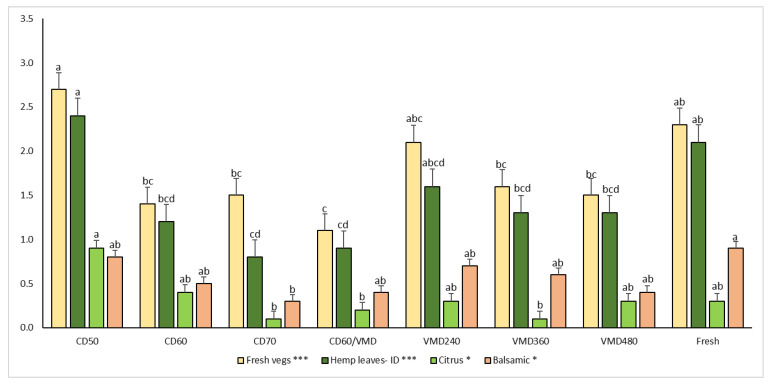
Sensory analysis of hemp leaves according to various drying methods. Bars, for a particular sensory descriptor (e.g., fresh vegs), with the same letter were not significantly different (*p* < 0.05) according to the HSD Tukey’s least significance difference test.

The fresh sample was taken as the control, and it is easy to see in Figure 2 that the samples with the closest aroma profile to this control were those dried using CD at 50 °C and VMD at 240, 360 and 480 W. This descriptive data agreed quite well with the data found in the ranking experiment, with CD at 50 °C and VMD at 240 and 360 W leading to samples with the highest aroma intensity and the more complex aroma profiles.

## 3. Materials and Methods

### 3.1. Plant Material

Leaves of hemp var. Henola amounting to 15 kg were collected on 15 October 2022 from a field in Oborniki Śląskie (16°55′ E, 51°18′ N), Poland. After being manually removed from the stalk, the leaves were combined and subjected to drying processes, followed by distillation and other chemical analyses. The moisture content of the material at the beginning of the study was 82%, which was assessed using an SPT-200 ZEAMIL vacuum dryer (Horyzont, Kraków, Poland). The drying processes were suspended when no changes in the sample masses were observed. Voucher specimens were deposited at the Department of Food Chemistry and Biocatalysis at Wroclaw University of Environmental and Life Sciences.

### 3.2. Drying Methods

Hemp leaves samples (60 g) were subjected to convective drying (CD), vacuum–microwave drying (VMD) as well as combined convective pre-drying and vacuum–microwave finishing drying (CD-VMD) according to the methods described by Kwaśnica et al. [14]. Briefly, CD was performed at 50, 60 and 70 °C using a dryer constructed at the Institute of Agricultural Engineering (Wroclaw University of Environmental and Life Sciences, Wroclaw, Poland), while VMD was conducted at 240, 360 and 480 W using an SM 200 dryer (Plazmatronika, Wroclaw, Poland). Drying conditions were determined based on the preliminary studies and literature review [14]. As previously proved in the studies on thyme [8] and marjoram [37], combined drying provides good results both in terms of reduced drying time and improved chemical properties of dried material due to the synergistic effect of drying methods used. Therefore, CD-VMD was performed, wherein the first part of drying was constituted by CD at 60 °C for 1 h followed by vacuum–microwave finishing drying at 360 W. All drying variants were performed until the same weight of the material was maintained during three consecutive weight measurements. The drying experiment was performed in two technological replications.

### 3.3. Drying Kinetics and Models

Drying kinetics were appointed on the basis of mass losses and illustrated as moisture ratio calculated using the simplified equation according to Calín-Sánchez et al. [38] (Equation (1)).

(1) $$MR=\frac{M_{\left(t\right)}}{M_{0}}$$

where *M*_(*t*)_ is the moisture ratio at the time of drying and *M*_0_ is the initial moisture content. Based upon drying kinetics, simple mathematical models were fitted to the experimental data using Table Curve 2D software. Drying was performed in monolayer in two technological repetitions and modified Page, logarithmic as well as Midilli models were used to describe drying kinetics (Table 8).

### 3.4. Sample Preparation and Chromatographical Analysis

#### 3.4.1. Volatile Compounds Analysis

##### Headspace Solid-Phase Microextraction (HS-SPME)

The extraction of VOCs from hemp leaves was carried out using the HS-SPME technique, using fiber (2 cm, DVB/CAR/PDMS, Supelco, Bellefonte, PA, USA). A weighed quantity of homogenized 0.02 g dry and 0.06 g fresh plant sample was placed in a headspace analysis vial with the addition of an internal standard of 25 µg 2-undecanone (Sigma-Aldrich, Steinheim, Germany). The prepared sample was incubated in a water bath for 10 min at 45 °C and then the volatile compounds absorbed on the fiber for 25 min. Analysis of the prepared sample was performed on a Varian CP-3800/Saturn 2000 (Varian, Walnut Creek, CA, USA) equipped with a Zebron ZB-5 column (30 m, 0.25 mm, 0.25 µm; Phenomenex, Torrance, CA, USA). Desorption of analytes from the fiber took 3 min in a 250 °C injector. The GC temperature program was programmed as follows: 40 °C hold for 3 min (initial temperature), then to 110 °C at a rate of 5 °C min^−1^ and to 270 °C min at a rate of 20 °C min^−1^. Scanning was set to range between 40 and 300 *m*/*z* in electron impact (EI) mode at 70 eV. The carrier gas was helium at a flow rate of 1.1 mL min^−1^ and with the split ratio of 1:20. The analysis was carried out in triplicate. Obtained results of SPME analyses are expressed as percentage of all identified compounds according to MS total ion current. Response factor for MS detector for each group of compounds was used for quantification.

##### Essential Oils

A Deryng apparatus was used to extract essential oils from hemp leaves. A weighed amount of fresh and dry plant material was transferred to a 250 mL round-bottom flask and poured over 100 mL of distilled water. The flask was placed on a heating bench and the contents brought to a boil. The process was carried out for 50 min and the essential oil was collected in a volume of 1 mL of cyclohexane, which contained 1 mg of 2-undecanone as internal standard (Sigma-Aldrich, Saint Louis, MO, USA). After distillation, the organic fraction was collected and stored at −18 °C until chromatographic analysis.

The profile of volatile compounds was determined using a gas chromatographer coupled to a mass spectrometer (Shimadzu GCMS QP 2020, Shimadzu, Kyoto, Japan). The column used for compound separation was a Zebron ZB-5 with the following dimensions: 30 m, 0.25 mm, 0.25 μm; Phenomenex, Torrance, CA, USA. The apparatus operated according to the following parameters during analyses: electron ionization mode at 70 eV, scanning in the range of 35 to 400 *m*/*z* in the option 2 scans s^−1^. The carrier gas was helium at a flow rate of 13.3 mL min^−1^ with a split of 1:10. The temperature program of the GC unit was programmed as follows: 50 °C (initial temperature) to 250 °C at a rate of 3 °C min^−1^ and kept for 3 min. The analysis was carried out in triplicate. Response factor for MS detector for each group of compounds was used for quantification. Results are expressed as mg per 100 g and percentage of identified EOs constituents.

#### 3.4.2. Cannabinoids, Sterols and Triterpenoids Analysis

Leaves with a mass of 100 mg were placed in an Eppendorf tube and 100 µg of cholesterol was also added to the sample to serve as an internal standard. The entire sample was flooded with a mixture of organic solvents (acetone:dichloromethane 1:1) and vigorously shaken. Samples were centrifuged on a bench-top centrifuge at 13,000 rpm and the extract, after filtration through a celite filter, was transferred to a GC vial. The extraction was performed twice. The organic fraction was evaporated on a vacuum evaporator under reduced pressure. An amount of 500 µL of pyridine and 100 µL of the derivatizing reagent N,O-bis (trimethylsilyl) trifluoroacetamide (BSTFA) were added to the samples. The derivatization process was carried out on a heating panel at 70 °C for 20 min. Analyses were prepared in triplicate.

Samples were also analyzed using Shimadzu GC-MS equipment (Shimadzu GCMS QP 2020, Shimadzu, Kyoto, Japan), equipped with a Zebron ZB-5 column (30 m, 0.25 mm, 0.25 µm; Phenomenex, Torrance, CA, USA). During the analysis, the camera operated in two modes: SCAN and SIM (3 scans s^−1^). For SCAN mode, the scan range was 50 to 650 *m*/*z*, while for SIM mode, the detector collected the following ion *m*/*z* values: 129, 203, 218, 303, 337, 343, 357, 367, 371, 386, 390, 394 and 491. The carrier gas flow rate, which was helium, was set at 0.98 mL min^−1^ with a 1:50 split. The injector was set at 250 °C. The program settings during the analysis were as follows: initial temperature 180 °C, then a rate of 5 °C min^−1^ to 280 for 7 min.

#### 3.4.3. Analysis of Obtained Data

The analysis and identification of compounds was based on three independent methods: (i) comparison of the calculated retention indices (RIs) using the retention index calculator with the values appearing in the NIST20 and Adams databases, (ii) comparison of the obtained spectra with the NIST20 and Adams databases, (iii) comparison of the retention indices of the compounds with authentic standards. The following software was used during the data analysis: GCMS Solution (v. 4.20), Spectrus Processor (v. S55S41) and AMDIS (v. 2.73).

### 3.5. Sensory Analysis

#### 3.5.1. Ranking Test

The samples with the highest and lowest intensity of “fresh hemp leaves-ID” were identified using a ranking test. This test is very useful in reducing the number of samples to be used in descriptive testing, by determining whether significant differences in the intensities of key attributes exist. Sixteen panelists (50% females and 50% males, with ages ranging from 24 to 55 years) ranked the intensity of “fresh hemp leaves-ID”. Between samples, crackers and water were available to clean the mouth.

#### 3.5.2. Descriptive Test

A trained panel (8 panelists, 50% female, ages 24–55 years) was used to rate the intensity of the key sensory descriptors of dried hemp leaves. These panelists can be considered highly trained, as they have over 1000 h of experience in describing dried products [21], and even hemp-based products [14]. Panel selection, training and validation were conducted according to the International Organization for Standardization ISO standard 8586-1 (1993) [42].

Descriptive sensory analysis (DSA) was used to define the aroma (perception of volatile compounds with the samples outside the mouth) profile of dried hemp leaves. To develop a proper lexicon (1 session of 90 min), panelists tried different hemp-based products to agree on the key descriptors to be used in a full description of the products under analysis (fresh and dried hemp leaves). The final lexicon consisted of 7 attributes: (i) fresh hemp-based products: fresh vegetables, hemp leaves-ID, citrus, balsamic and spicy; and (ii) dried hemp-based products: hay-woody and chamomile. Attributes previously found in dried hemp flowers such as cooked, earthy and burnt were not used because they were not identified in the samples under analysis. To have a fully objective use of the scale, ranging from 0 to 10 (0 = none or not perceptible intensity and 10 = extremely high intensity), reference products were prepared, with intensities similar to those of the samples under evaluation.

The sensory tests were conducted in normalized cabins with controlled temperature and illumination. Samples were prepared in odor-free plastic cups with lids (100 mL) and were left to equilibrate for 15 min at room temperature before serving. Then, samples were randomly served to panelists, using 3-digit codes. To avoid fatigue among the panelists, the 8 samples to be analyzed were divided in two groups of 4 and were analyzed in two independent sessions.

### 3.6. Statistical Analysis

Mathematical modelling was performed using Table Curve 2D v. 5.01 (Systat Software, Inc., San Jose, CA, USA). Models were fitted based on root mean square error (RMSE) and coefficient of determination *R*^2^. A one-way analysis of variance (ANOVA) was performed to analyze the experimental data obtained using STATISTICA v. 13.0 (StatSoft, Inc., Tulsa, OK, USA). For chromatographical analysis, Tukey’s test was used.

## 4. Conclusions

For the first time, a study was performed on the detailed analysis of phytochemical changes in hemp leaves subjected to microwave, convection and combined drying. The essential oil content in hemp leaves was measured at 166 mg 100 g^−1^ d.w. level. The most conservative result for the essential oil content maintenance methods was the CD60 VMD combined method, in which a loss of 63% of volatile fractions was observed. The least favorable (a loss of 83.68 and 83.45%) was observed in the VMD 240 and CD 70 variants. Sensory analysis showed that a temperature of 50 °C leads to the least change in the plant material, which is associated with higher consumer acceptance. We proved that none of the applied drying variants has a statistically significant effect on the quantitative and qualitative profiles of phytosterols and cannabinoids. No CBDA or THCA decarboxylation processes were observed in all the applied drying variants. This is the first result proving the thermal stability of the cannabinoid fraction in hemp leaves during the mild drying process.

## Figures and Tables

**Table 1:** Mathematical models applied to fit experimental data during drying of hemp leaves.

Model Name	Drying Conditions	Constants				Statistics	
		*a*	*k*	*b*		RMSE	*R* ^2^
Logarithmic	CD50	1.000	0.0301	0.0327		0.0102	0.9991
	CD60	1.000	0.0412	0.0217		0.0125	0.9987
	CD70	1.000	0.0525	0.0129		0.0121	0.9988
	VMD240	1.000	0.0936	0.0291		0.0181	0.9958
	VMD360	1.000	0.1111	0.0195		0.0154	0.9972
	VMD480	1.000	0.1595	0.0055		0.0816	0.9527
	CD60-VMD	0.115	0.3897	0.0395		0.0028	0.9966
		*a*	*k*	*B*	*C*	RMSE	*R* ^2^
Midilli	CD50	1.000	0.0288	0.9874	0.00015	0.0082	0.9994
	CD60	1.000	0.0307	1.0751	0.00017	0.0104	0.9991
	CD70	1.000	0.0403	1.0788	0.00018	0.0064	0.9997
	VMD240	1.000	0.0638	1.1228	0.00060	0.0143	0.9975
	VMD360	1.000	0.0930	1.0120	0.00071	0.0123	0.9984
	VMD480	1.000	0.0342	1.8377	0.00178	0.0113	0.9992
	CD60-VMD	0.155	0.4330	0.5643	0.01173	0.0027	0.9974
		*a*	*k*	*n*		RMSE	*R* ^2^
Modified Page	CD50	1.000	0.0332	0.9389		0.0218	0.9956
	CD60	1.000	0.0338	1.0367		0.0185	0.9970
	CD70	1.000	0.0436	1.0465		0.0136	0.9984
	VMD240	1.000	0.0806	1.0133		0.0277	0.9901
	VMD360	1.000	0.1087	0.9782		0.0193	0.9957
	VMD480	1.000	0.0393	1.7355		0.0245	0.9957
	CD60-VMD	0.155	0.5665	0.3242		0.0043	0.9959

**Table 2:** Maximum temperature (T_max_), time of drying (t_CD_, t_VMD_) and color of hemp leaves.

Drying Method	T_max_	t_CD_	t_VMD_	Color		
	°C	min	min	L *	a *	b *
CD50	50	300	-	43.27 ± 0.11 ^a,1^	−4.32 ± 0.89 ^a^	12.01 ± 0.31 ^a,c^
CD60	60	210	-	43.45 ± 0.2 ^a,d^	−3.21 ± 0.29 ^b^	12.53 ± 0.27 ^a^
CD70	70	150	-	44.08 ± 0.24 ^d^	−3.51 ± 0.24 ^b,c^	14.21 ± 0.22 ^d^
VMD240	50	-	76	41.29 ± 0.3 ^e^	−3.31 ± 0.1 ^b,c^	11.49 ± 0.09 ^b,c^
VMD360	50	-	48	42.93 ± 0.18 ^a,c^	−4.02 ± 0.14 ^a,c^	12.37 ± 0.13 ^a^
VMD480	59	-	22	42.09 ± 0.44 ^b^	−3.29 ± 0.26 ^b,c^	12.4 ± 0.19 ^a^
CD60-VMD	48	60	21	42.49 ± 0.64 ^b,c^	−3.88 ± 0.29 ^a,b,c^	11.18 ± 0.46 ^b^

^1^ Mean values followed by the same letter were not significantly different (*p* < 0.05) according to the HSD Tukey’s least significance difference test. CD—convective drying, VMD—vacuum–microwave drying, *L*—lightness, *a*—red/green, *b*—blue/yellow value. *—according to CIELab convention.

**Table 5:** Changes in essential oils profiles of hemp leaves according to various drying methods.

Compound	Fresh	CD50	CD60	CD70	240VMD	360VMD	480 VMD	CD60/VMD
		Content % ^1^						
Limonene	4.67 ^a,2^	0.46 ^c^	0.45 ^c^	0.17 ^d^	1.26 ^b^	1.06 ^b^	0.41 ^c^	0.49 ^c^
Caryophyllene	33.18 ^a^	20.76 ^b^	12.50 ^f^	10.19 ^g^	13.76 ^ef^	16.58 ^d^	14.32 ^e^	21.12 ^c^
Humulene	3.57 ^a^	9.96 ^g^	6.40 ^c^	4.85 ^b^	6.79 ^d^	7.80 ^e^	7.40 ^e^	8.28 ^f^
Caryophyllene oxide	4.65 ^a^	13.44 ^e^	11.38 ^cd^	8.92 ^b^	11.93 ^d^	10.21 ^c^	13.82 ^e^	11.18 ^cd^
Humulene epoxide II	8.25 ^a^	5.61 ^b^	4.57 ^d^	3.46 ^f^	4.94 ^c^	4.21 ^e^	5.72 ^b^	4.40 ^ed^
Caryophylla-4(12),8(13)-dien-5α-ol	2.02 ^a^	8.16 ^b^	12.14 ^c^	8.24 ^b^	9.80 ^b^	9.80 ^b^	8.75 ^b^	8.96 ^b^
14-hydroxy-*cis*-Caryophyllene	2.71 ^a^	5.81 ^b^	7.41 ^c^	5.87 ^b^	7.12 ^c^	6.19 ^b^	6.00 ^b^	6.00 ^b^
14-hydroxy-9-epi-*trans*-Caryophyllene	3.16 ^a^	5.36 ^b^	7.20 ^e^	5.43 ^b^	6.66 ^de^	5.63 ^bc^	6.23 ^cd^	5.57 ^bc^
TOTAL (mg/100 g) d.w.	166.13	28.13	31.15	27.51	27.12	47.75	48.02	59.95
% recovery of EOs	100	16.93	18.75	16.55	16.32	28.56	28.91	36.08

^1^ Calculated by peak area normalization according to internal standard; ^2^ values followed by the same letters are not statistically different (Tukey’s test, *p* > 0.05).

**Table 6:** Changes in cannabinoids and triterpenoids profiles of hemp leaves according to various drying methods.

Compound, TMS	ANOVA	Fresh	CD50	CD60	CD70	240VMD	360VMD	480 VMD	CD60/VMD
		Concentration (mg g^−1^) ^1^							
CBD	NS ^2^	2.19	1.98	1.87	1.88	1.89	2.01	2.08	1.95
CBC	NS	0.05	0.06	0.06	0.08	0.07	0.07	0.08	0.05
Δ^8^-THC-d_8_	NS	0.02	0.03	0.03	0.03	0.04	0.04	0.04	0.04
Δ^9^-THC-d_9_	NS	0.25	0.21	0.23	0.27	0.28	0.25	0.28	0.24
CBG	NS	0.09	0.07	0.08	0.10	0.08	0.14	0.10	0.09
CBN	NS	0.04	0.02	0.02	0.03	0.03	0.03	0.03	0.02
CBDA	NS	6.05	5.48	5.65	5.74	5.79	5.62	5.61	5.85
THCA	NS	0.63	0.59	0.67	0.60	0.55	0.48	0.49	0.52
CBGA	NS	0.19	0.20	0.23	0.20	0.17	0.19	0.16	0.14
TOTAL		9.51	8.64	8.84	8.93	8.90	8.83	8.87	8.90

^1^ Data are relative concentrations expressed per internal standard; ^2^ NS—not statistically different.

**Table 7:** Changes in sterols profiles of hemp leaves according to various drying methods.

Compound, TMS	ANOVA	Fresh	CD50	CD60	CD70	240VMD	360VMD	480 VMD	CD60/VMD
		Concentration (µg g^−1^) ^1^							
Campesterol	NS ^2^	97.90	100.37	86.70	92.27	93.22	108.87	107.33	104.11
Stigmasterol	NS	44.51	36.31	35.26	49.23	33.39	43.27	51.22	50.51
β-Sitosterol	NS	534.11	515.77	421.81	448.79	527.28	444.21	414.75	371.27
β-Amyrin	NS	65.96	40.68	60.41	64.33	49.50	60.41	65.12	56.53
Isofucosterol	NS	41.38	40.56	44.94	47.94	38.27	47.31	46.65	50.64
α-Amyrin	NS	61.07	65.91	72.09	73.80	67.42	70.45	73.15	73.26
Lupeol	NS	97.45	90.30	94.21	90.78	82.49	84.80	96.59	101.87
TOTAL		855.38	889.9	815.42	867.14	891.57	869.32	874.81	808.19

^1^ Data are relative concentrations expressed per internal standard; ^2^ NS—not statistically different.

**Table 8:** Mathematical models used in the study.

Model Name	Equation	References
Modified Page	$MR=a\cdot e^{-k\cdot t^{n}}$	[38,39]
Midilli	$MR=a\cdot e^{-k\cdot t^{B}}+C\cdot t$	[40]
Logarithmic	$MR=a\cdot e^{-k\cdot t}+B$	[16,41]

## Data Availability

Data is contained within the article or Supplementary Materials and available upon reasonable request from the corresponding author.

## Supplementary Materials

The following supporting information can be downloaded at: https://www.mdpi.com/article/10.3390/molecules28248089/s1, Data S1: EI-MS spectrum of unknown compound 1, Data S2: EI-MS spectrum of unknown compound 2, Data S3: Sample chromatogram of essential oil (liquid injection), Data S4: Sample chromatogram of aroma volatiles (SPME). Table S1. Changes in aroma profile of hemp leaves according to various drying methods. Table S2. Changes in essential oils profile of hemp leaves according to various drying methods. Table S3. Changes in cannabinoids and triterpenoids profile of hemp leaves according to various drying methods. Table S4. Changes in sterols profile of hemp leaves according to various drying methods. Tables S1–S4 are Table 4, Table 5, Table 6 and Table 7 from manuscript with ± SD.
