# Supplementary material for: Effect of Drying Methods on Chemical and Sensory Properties of Cannabis sativa Leaves
Source: Molecules. 2023 Dec 14;28(24):8089. doi: 10.3390/molecules28248089 (PMC10745367; doi:10.3390/molecules28248089)
Supplement: Supplementary file 1 [file molecules-28-08089-s001.zip › molecules-2718275-supplementary.pdf]

Suplementarny materials.

1. Data S1: EI-MS spectrum of unknown compound 1

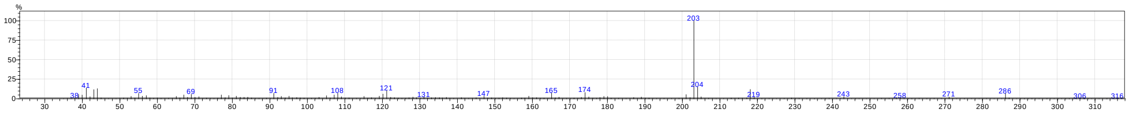

2. Data S2: EI-MS spectrum of unknown compound 2:

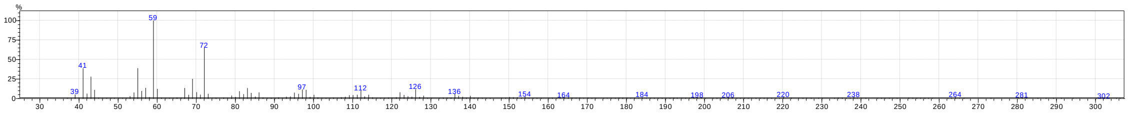

3. Data S3: Sample chromatogram of essential oil (liquid injection)

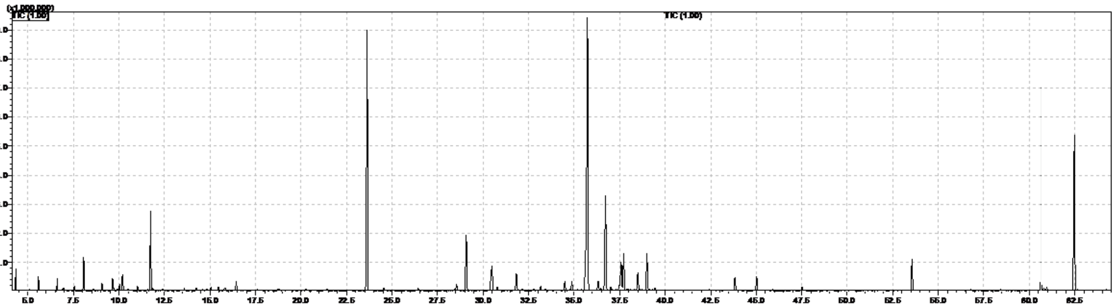

4. Data S4: Sample chromatogram of aroma volatiles (SPME)

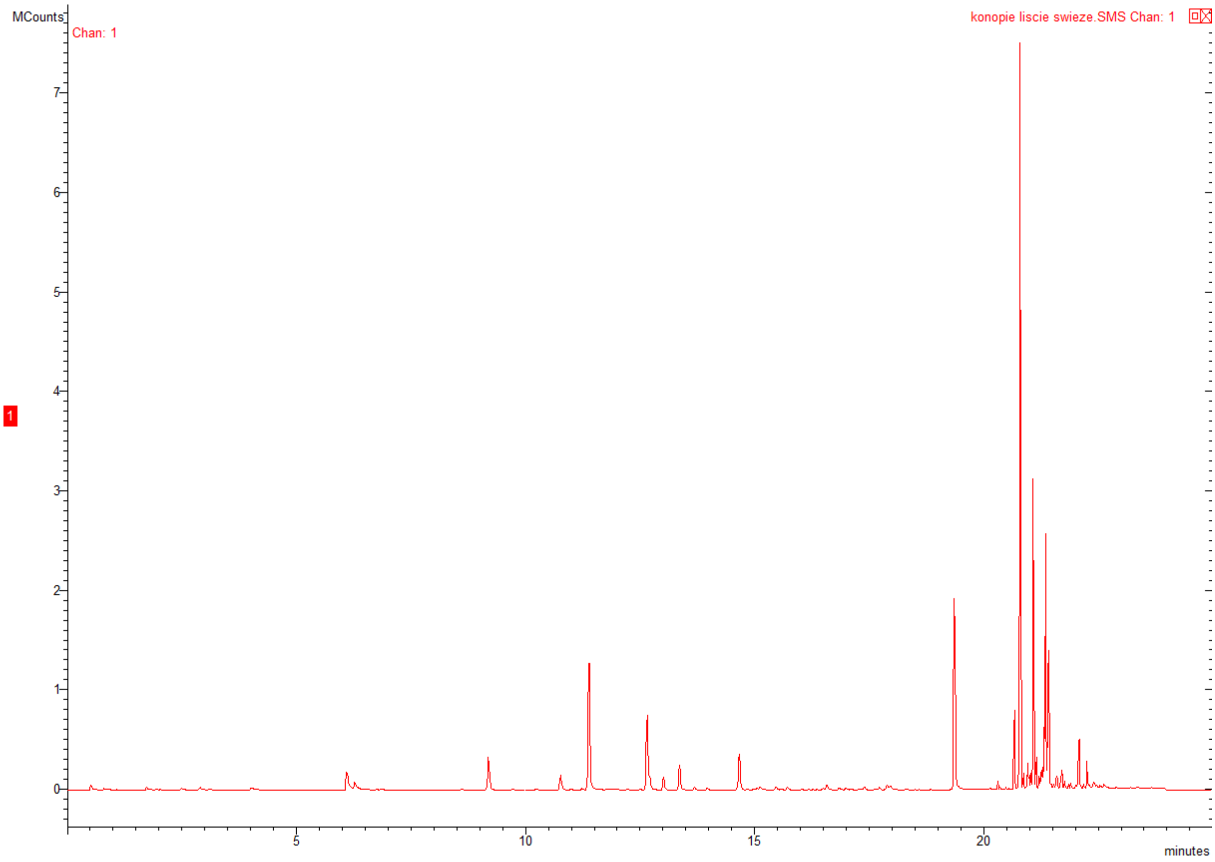

Table S1. Changes in aroma profile of hemp leaves according to various drying methods.

Compound	Fresh	CD50	CD60	CD70	240VMD	360VMD	480 VMD	CD60/VMD
		Content %[1]						
β-Myrcene	8.25±1.09[a,2]	11.96±1.56[b]	3.95±0.77[c]	1.62±0.89[f]	1.19±0.44[g]	1.30±0.26[h]	2.14±0.46[e]	2.47±0.41[d]
Limonene	5.47±0.98[a]	5.73±0.68[b]	1.95±0.65[e]	2.01±0.66[e]	0.95±0.12[g]	1.40±0.32[f]	4.78±0.51[c]	3.84±0.89[d]
Terpinolene	2.28±0.0.45[a]	1.32±0.24[c]	6.33±0.89[d]	3.34±0.41[b]	6.38±1.43[d]	9.53±1.45[e]	12.29±1.39[g]	10.36±1.06[f]
Isocaryophyllene	3.86±0.78[a]	1.95±0.21[f]	2.32±0.41[d]	2.09±0.16[e]	1.13±0.17[h]	1.64±0.98[g]	2.59±0.35[c]	3.08±0.86[b]
(E)-β-Caryophyllene	30.95±3.53[a]	18.03±1.86[d]	14.90±1.01[e]	13.24±1.06[f]	21.43±2.42[b]	20.69±1.78[c]	9.11±1.36[g]	7.10±1.36[h]
α-Humulene	11.22±1.42[a]	5.50±1.06[d]	5.26±1.08[e]	5.04±0.26[f]	7.45±1.45[b]	7.16±1.24[c]	4.11±0.98[g]	3.92±1.05[h]
β-Selinene	8.95±0.98[a]	1.21±0.24[h]	1.89±0.33[e]	2.05±0.11[d]	1.78±1.06[f]	1.50±0.98[g]	2.35±0.44[c]	2.84±0.76[b]
α-Selinene	7.00±1.04[a]	1.51±0.33[g]	2.07±0.21[e]	2.15±0.48[d]	1.19±0.13[h]	1.81±0.34[f]	2.33±0.56[c]	2.80±0.44[b]

[1] Calculated by peak area normalization according to internal standard; [2] Values followed by the same letters are not statistically different (Tukey's test, $p > 0.05$)

Table S2. Changes in essential oils profile of hemp leaves according to various drying methods.

Compound	Fresh	CD50	CD60	CD70	240VMD	360VMD	480 VMD	CD60/VMD
	Content %[1]							
Limonene	4.67±0.27[a,2]	0.46±0.06[c]	0.45±0.11[c]	0.170±0.06[d]	1.26±0.03[b]	1.06±0.13[b]	0.41±0.22[c]	0.49±0.09[c]
Caryophyllene	33.18±1.41[a]	20.76±0.99[b]	12.50±1.03[f]	10.19±1.41[g]	13.76±1.22[ef]	16.58±1.88[d]	14.32±1.29[e]	21.12±2.11[c]
Humulene	3.57±0.21[a]	9.96±1.01[g]	6.40±0.77[c]	4.85±0.35[b]	6.79±0.41[d]	7.80±0.49[e]	7.40±0.54[e]	8.28±0.48[f]
Caryophyllene oxide	4.65±0.41[a]	13.44±0.89[e]	11.38±0.99[cd]	8.92±0.56[b]	11.93±1.01[d]	10.21±1.21[c]	13.82±1.46[e]	11.18±1.03[cd]
Humulene epoxide II	8.25±0.22[a]	5.61±0.24[b]	4.57±0.46[d]	3.46±0.43[f]	4.94±0.51[c]	4.21±0.49[e]	5.72±1.87[b]	4.40±0.59[ed]
Caryophylla-4(12),8(13)-dien-5α-ol	2.02±0.11[a]	8.16±0.36[b]	12.14±0.78[c]	8.24±0.78[b]	9.80±0.43[b]	9.80±0.87[b]	8.75±0.98[b]	8.96±0.49[b]
14-hydroxy-*cis*-Caryophyllene	2.71±0.23[a]	5.81±0.16[b]	7.41±0.66[c]	5.87±0.66[b]	7.12±0.51[c]	6.19±0.66[b]	6.00±0.43[b]	6.00±0.78[b]
14-hydroxy-9-epi-*trans*-Caryophyllene	3.16±0.14[a]	5.36±0.14[b]	7.20±0.41[e]	5.43±0.89[b]	6.66±0.44[de]	5.63±0.48[bc]	6.23±0.47[cd]	5.57±0.44[bc]
TOTAL (mg/100g) d.w.[3]	166.13	28.13	31.15	27.51	27.12	47.75	48.02	59.95
% recovery of EOs	100	16.93	18.75	16.55	16.32	28.56	28.91	36.08

[1] Calculated by peak area normalization according to internal standard; [2] Values followed by the same letters are not statistically different (Tukey's test, $p > 0.05$)

Table S3. Changes in cannabinoids and triterpenoids profile of hemp leaves according to various drying methods.

Compound, TMS	ANOVA	Fresh	CD50	CD60	CD70	240VMD	360VMD	480 VMD	CD60/VMD
	Concentration (mg g^{-1})[1]								
CBD	NS[2]	2.19±0.27	1.98±0.12	1.87±0.13	1.88±0.24	1.89±0.65	2.01±0.14	2.08±0.12	1.95±0.33
CBC	NS	0.05±0.01	0.06±0.02	0.06±0.02	0.08±0.02	0.07±0.03	0.07±0.03	0.08±0.03	0.05±0.01
Δ^8-THC-d_8	NS	0.02±0.01	0.03±0.01	0.03±0.01	0.03±0.01	0.04±0.01	0.04±0.01	0.04±0.01	0.04±0.01
Δ^9-THC-d_9	NS	0.25±0.09	0.21±0.09	0.23±0.04	0.27±0.06	0.28±0.09	0.25±0.12	0.28±0.08	0.24±0.06
CBG	NS	0.09±0.01	0.07±0.03	0.08±0.02	0.10±0.04	0.08±0.02	0.14±0.09	0.10±0.04	0.09±0.03
CBN	NS	0.04±0.02	0.02±0.01	0.02±0.01	0.03±0.01	0.03±0.01	0.03±0.01	0.03±0.01	0.02±0.01
CBDA	NS	6.05±0.56	5.48±0.36	5.65±0.25	5.74±0.29	5.79±0.54	5.62±0.36	5.61±0.24	5.85±0.44
THCA	NS	0.63±0.09	0.59±0.08	0.67±0.12	0.60±0.08	0.55±0.14	0.48±0.24	0.49±0.07	0.52±0.06
CBGA	NS	0.19±0.08	0.20±0.09	0.23±0.07	0.20±0.15	0.17±0.05	0.19±0.11	0.16±0.04	0.14±0.03
TOTAL		**9.51**	**8.64**	**8.84**	**8.93**	**8.90**	**8.83**	**8.87**	**8.90**

[1] Data are relative concentrations expressed per internal standard; [2] NS – not statistically different.

Table S4. Changes in sterols profile of hemp leaves according to various drying methods.

Compound, TMS	ANOVA	Fresh	CD50	CD60	CD70	240VMD	360VMD	480 VMD	CD60/VMD
		Concentration (μg g^{-1})[1]							
Campesterol	NS[2]	97.90±1.43	100.37±3.13	86.70±2.18	92.27±2.47	93.22±3.27	108.87±3.46	107.33±3.57	104.11±4.12
Stigmasterol	NS	44.51±1.27	36.31±1.22	35.26±1.05	49.23±1.47	33.39±1.89	43.27±1.56	51.22±2.54	50.51±3.44
β-Sitosterol	NS	534.11±17.32	515.77±15.26	421.81±13.27	448.79±15.28	527.28±19.20	444.21±18.26	414.75±17.29	371.27±18.26
β-Amyrin	NS	65.96±2.48	40.68±1.45	60.41±2.10	64.33±1.99	49.50±1.79	60.41±2.48	65.12±4.99	56.53±2.56
Isofucosterol	NS	41.38±2.37	40.56±1.88	44.94±1.24	47.94±2.37	38.27±2.18	47.31±2.66	46.65±4.01	50.64±3.31
α-Amyrin	NS	61.07±2.11	65.91±1.36	72.09±1.56	73.80±3.11	67.42±2.14	70.45±3.56	73.15±4.19	73.26±4.28
Lupeol	NS	97.45±1.77	90.30±2.87	94.21±3.99	90.78±4.37	82.49±3.19	84.80±3.59	96.59±3.89	101.87±5.22
TOTAL		855.38	889.9	815.42	867.14	891.57	869.32	874.81	808.19

[1] Data are relative concentrations expressed per internal standard; [2] NS – not statistically different.
